# Prevalence and types of prescribing errors made by non-medical prescribers in health care settings globally: a systematic review protocol

**DOI:** 10.11124/JBIES-25-00015

**Published:** 2026-02-10

**Authors:** Saija Koskiniemi, Tiina Syyrilä, Laura Jukarainen, Bryony Dean Franklin, Minna Mykkänen, Virpi Jylhä, Marja Härkänen

**Affiliations:** 1 Department of Nursing Science, University of Eastern Finland, Kuopio, Finland; 2 JBI Finnish Centre for Evidence-Based Health Care, Helsinki, Finland; 3 School of Pharmacy, University College London, London, UK; 4 NIHR North West London Patient Safety Research Collaboration, London, UK; 5 Wellbeing Services County of North Savo, Kuopio, Finland; 6 Research Centre for Nursing Science and Social and Health Management, Kuopio University Hospital, Wellbeing Services County of North Savo, Kuopio, Finland

**Keywords:** errors, medication safety, non-medical prescriber, patient safety, prescribing

## Abstract

**Objective:**

The aim of this systematic review will be to establish the prevalence and types of prescribing errors made by non-medical prescribers in health care.

**Introduction:**

Prescribing errors are common, but the existing literature mostly focuses on medical prescribers. In many countries, legislation allows non-medical health care professionals to prescribe; however, there has been no systematic review of non-medical prescribing errors.

**Eligibility criteria:**

This review will include quantitative studies of all designs investigating the prevalence of prescribing errors related to any disease, medication, or patient group made by non-medical prescribers in all health care settings globally.

**Methods:**

This systematic review will include a Study Within a Review. We will use JBI methodology for systematic reviews, particularly the guidelines for prevalence estimate reviews. An information specialist will conduct standard database searches using PubMed, CINAHL (EBSCOhost), Scopus, and Web of Science Core Collection. Two researchers will perform a parallel search using artificial intelligence software. Studies found through standard database searches and those found using artificial intelligence will be included if the 2 researchers independently decide that they meet the eligibility criteria after full-text screening. A comparison of the standard database search and artificial intelligence search will be reported in a separate Study Within a Review. Two researchers will independently conduct the critical appraisals and data extraction. A meta-analysis will be conducted, if possible, otherwise, a narrative synthesis will be used.

**Review registration:**

PROSPERO CRD42024616617

## Introduction

Medication errors are a serious but avoidable problem in health care. Worldwide, the annual cost of medication errors has been estimated to be US$42 billion.^1^ Medication errors include errors in administration, prescribing, transcribing, dispensing, and monitoring practices.^1^ Prescribing errors are a global problem, with the median error rate being 7% to 32% of medication orders, as reported in previous systematic reviews on paper-based prescribing.^2^,^3^ While prescribing errors have been extensively studied among physicians,^4^-^9^ less is known about the prevalence and types of errors made by non-medical prescribers. Definitions of prescribing errors vary across studies; consequently, investigating the prevalence of prescribing errors is challenging.^10^,^11^

Prescribing errors most commonly involve incorrect dosage, strength,^2^,^10^ or medication selection, and are most commonly related to antibiotics and analgesics,^11^ which may reflect these being the most commonly used groups of drugs. Prescriber-related factors, such as insufficient knowledge, prescribing skills, and experience, have been found to influence in-hospital prescribing errors in a systematic review.^12^ The review did not consider that, currently, prescribers’ backgrounds can vary significantly, with prescribers being either medical or non-medical.

Dentists and physicians are considered medical prescribers. Other health care professionals with a qualification to prescribe are generally referred to as non-medical prescribers with legal prescribing rights, such as nurses, pharmacists, and occupational therapists.^13^ According to Ecker *et al*.,^13^ of 117 countries in a scoping review, 99 have some non-medical prescribing practices, although prescribing rights and levels vary. Non-medical prescribers may have full or nearly full prescribing rights, have more limited prescribing rights for particular medicines, or can continue medicines initiated by another prescriber.^14^ Independent prescribing refers to the practice of the prescriber autonomously prescribing medications. Prescribers with supplementary prescribing rights can, for example, renew prescriptions or prescribe under the supervision of a medical prescriber.^14^ Unlike independent and supplementary prescribing, health care professionals following patient group directions do not prescribe medicines. Patient group directions enable the administration of specific patient groups in specific situations using specified medicines.^15^

As the number of non-medical prescribers is increasing, it is essential to understand the safety and quality of non-medical prescribing practices. Reported prescribing error rates vary significantly across non-medical professions, but also across different counties. For example, in the critical care setting in the UK, the prescribing error rate of advanced nurse practitioners (0.6%) was statistically significantly lower than that of consults (6.8%) and other medical prescribers (3.4%), where the overall error rate was 2.8%.^16^ Another study conducted in critical care in the UK found a low prescribing error rate (0.18%) among pharmacists.^17^ The third study from the UK supports the finding that pharmacists have a low prescribing error rate (0.7%), also across the acute hospital setting.^18^ In contrast, neonatal nurse practitioners’ prescribing error rate was 16%, but still remarkably lower than pediatric residents’ error rate (39%) in the US.^19^ In digital health services, nurse practitioners’ error rate was lower (14.22%) compared to general practitioners (21.37%) in Australia.^20^ Current evidence of non-medical prescribing errors remains fragmented, as no other systematic review has focused on this topic. Thus, a systematic review is necessary to compile the existing knowledge, identify research gaps, and guide future policy and practice regarding non-medical prescribing.

A preliminary search of PROSPERO, PubMed, and *JBI Evidence Synthesis* was conducted, and no current or in-progress systematic review on the topic were identified. Prior systematic reviews have focused on the prevalence and incidence of prescribing errors with high-risk medicines in hospitals^5^ and prescribing errors in handwritten prescriptions in primary or secondary care^21^ made by medical prescribers. Systematic reviews of prescribing errors among adults and children as hospital inpatients using paper prescriptions,^3^ types and causes of prescribing errors associated with computerized provider order entry systems,^22^ and factors influencing prescribing errors in hospitals^23^ did not define whether prescribers were medical or non-medical. None of the systematic reviews have focused on the prevalence and incidence of prescribing errors made by non-medical prescribers.

This review’s objective is to establish the prevalence and types of prescribing errors made by non-medical prescribers in health care.

## Review questions

- What is the prevalence of prescribing errors made by non-medical prescribers in all health care settings globally?
- What types of prescribing errors are made by non-medical prescribers in all health care settings globally?

## Eligibility criteria

### Population

The population of this review will include non-medical prescribers, such as nurses, pharmacists, physiotherapists, occupational therapists, paramedics, optometrists, podiatrists, radiographers, and other allied health professionals. Medical prescribers, including physicians, dentists, and medical or dental students, will be excluded. All levels of non-medical prescribing (independent and supplementary) will be included. *Independent prescribing rights,* therefore, refer to a non-medical prescriber autonomously prescribing a medicine. In contrast, *supplementary rights* refer to a non-medical prescriber continuing a medicine initiated by another prescriber, renewing a prescription, prescribing under supervision, or prescribing limited medicines. A combination of independent and supplementary non-medical prescribing rights may also be included. Non-medical health professionals who follow patient group directions will be excluded.

### Condition

The condition of this review will include prescribing errors related to any disease, medication, or patient group. Different definitions of prescribing errors have made synthesis in systematic reviews more difficult.^3^,^9^ In this study, we will use the definition by Dean *et al*.^10^ of *prescribing error*, defined as an unintentional result of a prescribing decision or prescription writing process that causes a “reduction in the probability of treatment being timely and effective”^(p.235)^ or causes an increase in the risk of harm [to the patient] when compared with generally accepted practice.”^(p.235)^ The included studies do not have to cite this definition precisely; however, the definition must be conceptually similar. Dean *et al*.^10^ present scenarios for situations that meet or do not meet their definition of a prescribing error. We will only consider studies that include descriptions of prescribing errors or when descriptions meet situations mentioned in the paper by Dean *et al.*^10^ (“Situations that should be included as prescribing errors”^[p.236]^ or “Situations that may be considered prescribing errors, depending on the individual clinical situation”^[p.236]^). The error types, with examples, are available in Appendix I. If a study does not include descriptions of prescribing errors or none of the described prescribing errors meet the situation described by Dean *et al*.,^10^ it will be excluded. All period, point, and lifetime prevalence studies will be accepted.

### Context

All health care settings, including digital services, in all countries will be included.

### Types of studies

Research papers will be eligible if they describe observational and experimental study designs reporting the prevalence or incidence of prescribing errors among non-medical prescribers. Studies that include medical and non-medical prescribing errors will be included if they allow the data to be extracted separately for non-medical prescribers. Studies on medication errors more generally will be included if it is possible to separate the data relating to prescribing errors. Studies based on self-reporting of errors will be excluded unless the number of studies using other methods for identifying prescribing errors is too few (fewer than 5 studies) for analysis. Gray literature, such as dissertations, theses, government reports, and conference papers, will also be excluded.

## Methods

The proposed systematic review will be conducted in accordance with the JBI methodology for systematic reviews, particularly following the guidance for prevalence estimate reviews.^24^,^25^ The review will be reported in accordance with the Preferred Reporting Items for Systematic Reviews and Meta-Analyses (PRISMA) guidelines.^26^ The review protocol has been registered in PROSPERO (CRD42024616617). Alongside this review, a Study Within a Review (SWAR)^27^ will be conducted to explore the suitability of using artificial intelligence (AI tools when searching for scientific articles to include in systematic reviews as an adjunct to the standard database search process. The SWAR is registered in the SWAR Store (registration number: SWAR43).^28^

### Search strategy

**Figure 1:**
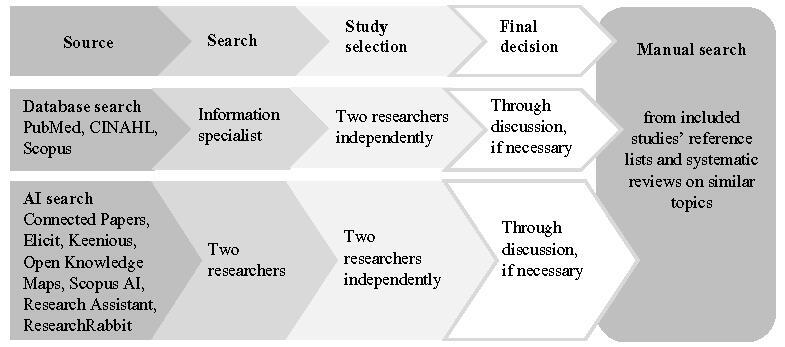
Data sources and study selection

The search strategy will aim to locate peer-reviewed published studies. The searches will be conducted separately but in parallel, using a standard database search and an AI search (Figure 1). The search strategy has been developed together with an information specialist. Studies published in all languages and for all dates will be accepted.

To conduct the database search, a 3-step search strategy will be used. First, an initial limited PubMed search was undertaken in February 2024 to identify articles on the topic. Second, the text words contained in the titles and abstracts of relevant articles and the index terms used to describe the articles were used to develop a full search strategy for PubMed, CINAHL(EBSCOhost), Web of Science Core Collection, and Scopus. A sample search strategy for CINAHL (EBSCOhost) is available in Appendix II. The search strategy, including all identified keywords and index terms, will be adapted for each included database and information source. The information specialist will conduct the final search in the databases. Third, the references of studies included in this review and systematic reviews on similar topics will be screened for additional studies.

To conduct the AI search, 2 researchers will use free versions of Connected Papers (Connected Papers, Israel), Elicit (Ought, SF, USA), Keenious (Keenious, Norway), Open Knowledge Maps (Open Knowledge Maps, Austria), Scopus AI (Elsevier, Singapore), Research Assistant (Clarivate, UK), and ResearchRabbit (ResearchRabbit, WA, USA).

### Study selection

Study selection from the results of the standard database search and the AI search will be made separately, but in parallel. Following the database search, all identified citations will be collated and uploaded into Covidence (Veritas Health Innovation, Melbourne, Australia), where duplicates will be removed. Subsequently, citations will be imported into the JBI System for the Unified Management, Assessment and Review of Information (JBI SUMARI; JBI, Adelaide, Australia).^29^ Following a pilot test, 2 reviewers will independently screen all titles and abstracts against the eligibility criteria. Before commencing the screening, however, both reviewers will also familiarize themselves with the definition and examples of prescribing errors,^3^ and only studies using that definition will be included. The same 2 reviewers will independently assess the full texts of selected citations in detail against the eligibility criteria. Reasons for excluding papers at full-text screening will be recorded and reported. Any disagreements between the reviewers at each stage of the selection process will be resolved through discussion or with a third independent reviewer. The search results and the study inclusion process will be reported in full in the final systematic review and presented in a PRISMA flow diagram.^26^ All papers passing the study selection process described here will be included, whether they are identified via the standard database search or the AI search alone, or both. Detailed results of the AI search will be reported in a separate SWAR article.

### Assessment of methodological quality

After a pilot test, 2 reviewers will independently conduct critical appraisal of eligible studies at the study level for methodological quality using the standardized critical appraisal instrument from JBI for prevalence studies^24^ in JBI SUMARI.^29^ The appraisal instrument will not be chosen based on the study design, but to evaluate the studies’ methodological rigor in relation to examining the prevalence of prescribing errors.^30^

Articles reporting incidence will be evaluated using the JBI critical appraisal tools based on the type of study design, including randomized controlled trials,^31^ quasi-experimental,^32^ cohort,^33^ and analytical cross-sectional studies.^33^ Authors of papers will be contacted via email to request missing or additional data for clarification, where required, and then followed up once. Any disagreements that arise will be resolved through discussion between the 2 reviewers. All studies, regardless of their methodological quality, will undergo data extraction and synthesis. The critical appraisal results will be reported descriptively and in a table.

### Data extraction

Two reviewers will independently extract the data from the included studies using the standardized data extraction tool for prevalence and incidence available in JBI SUMARI.^29^ The extracted data will include specific details about the source of reference, context, data collection date in each study, populations, study methods, prevalence, incidence, and types of prescribing errors. In addition to the fields in the standardized data extraction tool, we will extract information on countries, the profession of prescribers, the level and details of prescribing rights (eg, independent, supplementary), type of prescribing error, prevalence and incidence of prescribing errors, how errors were detected, prescribing error situations (eg, admission, discharge), and type of prescription system (eg, electronic, paper).

The data extraction tool will be piloted on 5 studies by the reviewers. Authors of papers will be contacted to request missing or additional data, where required, and then followed up once. Three authors will independently test the data extraction form before using it. Any disagreements that arise between the reviewers will be resolved through discussion.

### Data synthesis

Where possible, the studies will be pooled in a statistical meta-analysis using JBI SUMARI.^29^ The total number of prescribing errors and non-medical prescribers will be calculated across all studies. The pooled prevalence of prescribing errors will be expressed as a proportion with 95% CI. The data will be transformed using the Freeman–Turkey transformation. The heterogeneity of the studies will be assessed using the standard *I^2^* test. The *I^2^* test scores of 25%, 50%, and 75% will be considered low, moderate, and high. Subcategories (eg, level of prescribing right, patient group, country) will be examined, if feasible. Publication bias will be investigated with an appropriate statistical test (eg, Egger test) and the Doi plot generated using R software v.3.6.1. (R Foundation for Statistical Computing, Vienna, Austria).

Narrative synthesis will be conducted where statistical pooling is not possible. The results will then be presented in narrative format, including tables and figures. The data synthesis will focus on grouping error rates and types based on the studies’ similarities. Studies will be grouped into errors occurring in inpatient, outpatient, long-term, and home care, as well as in digital health services (eg, telemedicine, phone- or chat-based appointments). The studies will be further grouped according to the level of the prescribing right; the type of error; patient group; and prescription system type. If possible, information on prescribing error situations (eg, admission, discharge) will be collected and used in grouping. Further grouping will be considered based on the extracted data from the included studies. The synthesized findings will be compared with the findings of systematic reviews of prescribing errors made by medical prescribers.^2^,^9^

## Funding

The Wellbeing Service County of North Savo, Research Centre for Nursing Science and Social and Health Management, funded an information specialist. BDF is funded by the National Institute for Health and Care Research (NIHR) North West London Patient Safety Research Collaboration. The views expressed are those of the authors and not necessarily those of the NIHR or the Department of Health and Social Care. The Research Council of Finland funds TS and MH.

## Acknowledgments

In the memory of the information specialist, Maarit Putous (University of Eastern Finland), whose original idea was to combine standard database searches and use AI tools in this systematic review. Information specialist, Heikki Laitinen (University of Eastern Finland), for planning our search strategy.

## Author contributions

Planning of the review and search strategy, and writing the protocol: all authors. Pilot search: SK and an information specialist. All authors have approved the final version.

## Appendix I: Draft data extraction form

**Table:**

Authors	
Year of publication	
Date of data extraction	
Journal	
Country	
Study method	
Data collection date	
Health care context	
Number of non-medical prescribers	n =
Profession of non-medical prescribers	
Level of prescribing right	Details (eg, independent, supplementary)
Type of prescribing error (according to Dean *et al*.’s^10^ definition)	1. Inappropriate medication for the patient
	• The medication was contraindicated (eg, warfarin is prescribed to a patient with active gastrointestinal bleeding).
	• No indication for prescribed medication (eg, prescribing amoxicillin for viral pharyngitis).
	• The patient had a known allergy to the medication (eg, prescribing ibuprofen to a patient with a known NSAID allergy).
	• The medication had a potential drug interaction (eg, simvastatin was prescribed together with clarithromycin).
	• The medication and/or the dose were inappropriate for the patient’s renal function (eg, metformin was prescribed to a patient with severe renal impairment).
	• Incorrect dose (eg, the dose below recommended for the patient’s clinical condition, overdose with narrow therapeutic index, no dose adjustment after abnormal levels).
	• Continuing a drug despite adverse reaction (eg, clarithromycin was continued despite the patient getting a rash).
	• Unnecessary duplicate medication when one was sufficient (eg, prescribing both ibuprofen and naproxen for pain).
	2. Failure in prescription writing
	• Drug, dose, or route not as intended
	• Omission of indicated medication for a clinical condition
	• Illegible prescription
	• Misspelled medication (eg, amoxcillin was prescribed instead of amoxicillin)
	• Drug name written using abbreviations or non-standard nomenclature (eg, MTX was prescribed instead of methotrexate)
	• Ambiguous medication order
	• Prescribed “1 tablet” when multiple strengths exist
	• Prescribed “milligrams” when “micrograms” was intended
	• Dose cannot be administered using available dosage forms
	• Dose regimen not recommended for the prescribed formulation (eg, medication needing multiple daily doses was prescribed once-daily dosing)
	• Route of administration omitted for drug with multiple routes
	• Prescription continued longer than necessary (eg, amoxicillin prescribed for 10 days instead of the recommended 5)
	• Duration of intermittent IV infusion not specified
	• Timing in relation to meals was not specified
	• Prescriber’s signature omitted
	3. Failure regarding transcription
	• Omission of a pre-admission medication upon hospital admission (eg, the patient’s antihypertensive medication was not continued upon hospital admission)
	• Continuation of a general practitioner’s prescribing error upon hospital admission
	• Incorrect transcription when rewriting the medication chart (eg, insulin was transcribed as 10 units instead of the prescribed 1 unit)
	• Discharge prescription unintentionally deviated from the inpatient chart (eg, the discharge prescription omitted the patient’s regular anticoagulant medication that was continued during hospitalization)
	• Medication order upon admission deviates from pre-admission prescription (eg, the patient’s pre-admission antidepressant was replaced with a different preparation without a documented rationale)
Prevalence of prescribing errors	
Incidence of prescribing errors	
Prescribing error situation	eg, admission or discharge
How errors were detected	
Type of prescription system	eg, electronic or paper

IV, intravenous; NSAID, nonsteroidal anti-inflammatory drug.

## Appendix II: Search strategy

**Table:**

CINAHL (EBSCOhost)		
Search conducted: June 10, 2025		
#	Query	Records retrieved
1	Non-Medical Prescribers [MeSH] OR Non-Medical Prescribing [MeSH] OR Supplementary Prescribing [MeSH]	96
2	(nonmedical OR “non medical” OR “not medical” OR “non physician*” OR “not physician*” OR “non dentist*” OR nurse* OR “nursing staff” OR “nursing personnel” OR pharmacist* OR “pharmac* staff” OR “pharmac* personnel” OR physiotherapist* OR “physical therapist*” OR “occupational therapist*” OR “allied health personnel” OR “allied health staff” OR “allied health occupation*” OR “allied health professional” OR paramedic* OR optometrist* OR podiatrist* OR radiographer*) N5 prescri*	14,680
3	#1 OR #2	14,680
4	prescri* N5 (error* OR erroneous* OR flaw* OR fault* OR mistake* OR incorrect* OR false OR wrong)	1659
5	Inappropriate Prescribing [MeSH] OR Over Prescribing [MeSH]	4015
6	#4 OR #5	5594
7	Prevalence [MeSH] OR Prevalence Studies [MeSH] OR Incidence [MeSH]	186,963
8	Prevalence OR Incidence	511,629
9	#7 OR #8	511,629
10	#3 AND #6 AND #9	**767**

## References

[1] World Health Organization. Medication without harm: global patient safety challenge on medication safety [internet]. WHO; 2017 [cited 2024 Dec 1]. https://www.who.int/initiatives/medication-without-harm.

[2] Alhomoud FK Alnemari W Alfahmi H Alhomoud F Cheema E. Incidence and prevalence of prescribing errors in Saudi Arabia: a systematic study. Int J Pharm Pharm Sci 2016;8(12):194.

[3] Lewis PJ Dornan T Taylor D Tully MP Wass V Ashcroft DM. Prevalence, incidence and nature of prescribing errors in hospital inpatients: a systematic review. Drug Saf 2009;32(5):379-89. 19419233 10.2165/00002018-200932050-00002

[4] Simegn W Weldegerima B Seid M Zewdie A Wondimsigegn D Abyu C Assessment of prescribing errors reported by community pharmacy professionals. J Pharm Policy Pract 2022;15(1):62. 36243738 10.1186/s40545-022-00461-9 PMC9569042

[5] Cope LC Abuzour AS Tully MP. Non-medical prescribing: where are we now? Ther Adv Drug Saf 2016;7(4):165-72. 27493720 10.1177/2042098616646726 PMC4959632

[6] Canales-Siguero MD García-Muñoz C Caro-Teller JM Piris-Borregas S Martín-Aragón S Ferrari-Piquero JM Electronic prescribing in the neonatal intensive care unit: analysis of prescribing errors and risk factors. J Med Syst 2025;49(1):26. 39964641 10.1007/s10916-025-02161-8

[7] Zaal RJ van der Kaaij ADM Evenhuis HM van den Bemt PMLA. Prescription errors in older individuals with an intellectual disability: prevalence and risk factors in the Healthy Ageing and Intellectual Disability Study. Res Dev Disabil 2013;34(5):1656-62. 23501585 10.1016/j.ridd.2013.02.005

[8] Salam M Al Anazi M Al-Jeraisy M. Prevalence and predictors of antibiotic prescription errors in an emergency department, Central Saudi Arabia. Drug Healthc Patient Saf 2015;7:103-11. 26082662 10.2147/DHPS.S83770 PMC4461133

[9] Babatunde KM Akinbodewa AA Akinboye AO Adejumo AO. Prevalence and pattern of prescription errors in a Nigerian kidney hospital. Ghana Med J 2016;50(4):233-7. 28579629 10.4314/gmj.v50i4.6 PMC5443671

[10] Dean B Barber N Schachter M. What is a prescribing error? Qual Health Care 2000;9(4):232-7. 11101708 10.1136/qhc.9.4.232 PMC1743540

[11] Yikna BB Abay WA Awgichew SY. Medicine prescribing practices and prescription errors evaluations at outpatient department in Debre Berhan Comprehensive Specialized Hospital, Amhara Regional State, Ethiopia. J Pharm Pract 2025;38(1):81-92. 39115938 10.1177/08971900241273176

[12] Franklin BD Reynolds M Shebl NA Burnett S Jacklin A. Prescribing errors in hospital inpatients: a three-centre study of their prevalence, types and causes. Postgrad Med J 2011;1033(87):739-45. 10.1136/pgmj.2011.117879 21757461

[13] Ecker S Joshi R Shanthosh J Ma C Webster R. Non-medical prescribing policies: a global scoping review. Health Policy 2020;124(7):721-6. 32471762 10.1016/j.healthpol.2020.04.015

[14] Maier CB. Nurse prescribing of medicines in 13 European countries. Hum Resour Health 2019;17(1):95. 31815622 10.1186/s12960-019-0429-6 PMC6902591

[15] National Institute for Health and Care Excellence. Patient group directions [internet]. NICE; 2017 [cited 2025 Jan 4]. Available from: https://www.nice.org.uk/guidance/mpg2/resources/patient-group-directions-pdf-1779401941189.

[16] Carberry M Connelly S Murphy J. A prospective audit of a nurse independent prescribing within critical care. Nurs Crit Care 2013;18(3):135-41. 23577948 10.1111/j.1478-5153.2012.00534.x

[17] Cross VJ Parker JT Law Min MYL Bourne RS. Pharmacist prescribing in critical care: an evaluation of the introduction of pharmacist prescribing in a single large UK teaching hospital. Eur J Hosp Pharm 2018;25(e1):e2-e6. 10.1136/ejhpharm-2017-001267 PMC6457156 31157059

[18] Turner E Kennedy MC Barrowcliffe A. An investigation into prescribing errors made by independent pharmacist prescribers and medical prescribers at a large acute NHS hospital trust: a cross-sectional study. Eur J Hosp Pharm 2021;28(3):149-53.

[19] Brown CL Garrison NA Hutchison AA. Error reduction when prescribing neonatal parenteral nutrition. Am J Perinatol 2007;24(7):417-27. 17624817 10.1055/s-2007-984404

[20] Talay L Vickers M Lu D. Nurse practitioner and general practitioner error rates in a large digital health service: a retrospective cohort analysis. Nurs Rep 2024;14(4):3407-16. 39585137 10.3390/nursrep14040246 PMC11587444

[21] El Abdouni S Kalfsvel LS Rietdijk WJR van der Kuy H van Rosse F. Differences in prescribing errors between electronic prescribing and traditional prescribing among medical students: a randomized pilot study. Br J Clin Pharmacol 2024;91(8):2109-18. 38520277 10.1111/bcp.16053 PMC12304864

[22] Brown CL Mulcaster HL Triffitt KL Sittig DF Ash JS Reygate K A systematic review of the types and causes of prescribing errors generated from using computerized provider order entry systems in primary and secondary care. J Am Med Inform Assoc 2017;24(2):432-40. 27582471 10.1093/jamia/ocw119 PMC7651904

[23] Mahomedradja RF Schinkel M Sigaloff KCE Reumerman MO Otten RHJ Tichelaar J Factors influencing in-hospital prescribing errors: a systematic review. Br J Clin Pharmacol 2023;89(6):1724-35. 36805648 10.1111/bcp.15694

[24] Munn Z Moola S Lisy K Riitano D Tufanaru C. Systematic reviews of prevalence and incidence. In: Aromataris E Lockwood C Porritt K Pilla B Jordan Z, editors. JBI Manual for Evidence Synthesis [internet]. JBI; 2024 [cited 2025 Jan 4]. Available from: https://synthesismanual.jbi.global.

[25] Migliavaca CB Stein C Colpani V Barker TH Munn Z Falavigna M How are systematic reviews of prevalence conducted? A methodological study. BMC Med Res Methodol 2020;20(1):96. 32336279 10.1186/s12874-020-00975-3 PMC7184711

[26] Page MJ McKenzie JE Bossuyt PM Boutron I Hoffmann TC Mulrow CD The PRISMA 2020 statement: an updated guideline for reporting systematic reviews. BMJ 2021;372:n71. 33782057 10.1136/bmj.n71 PMC8005924

[27] Devane D Burke NN Treweek S Clarke M Thomas J Booth A Study within a review (SWAR). J Evid Based Med 2022;15(4):328-32. 36513956 10.1111/jebm.12505 PMC10107874

[28] The Northern Ireland Hub for Trials Methodology Research. SWAR store [internet]. MRC; 2024 [cited 2024 Oct 15]. Available from: https://www.qub.ac.uk/sites/TheNorthernIrelandNetworkforTrialsMethodologyResearch/SWATSWARInformation/Repositories/SWARStore/.

[29] Munn Z Aromataris E Tufanaru C Stern C Porritt K Farrow J The development of software to support multiple systematic review types: the JBI System for the Unified Management, Assessment and Review of Information (JBI SUMARI). Int J Evid Based Healthc 2019;17(1):36-43. 30239357 10.1097/XEB.0000000000000152

[30] Migliavaca CB Stein C Colpani V Munn Z Falavigna M, Prevalence Estimates Reviews – Systematic Review Methodology Group (PERSyst). Quality assessment of prevalence studies: a systematic review. J Clin Epidemiol 2020;127: 59-68. 32679313 10.1016/j.jclinepi.2020.06.039

[31] Barker TH Stone JC Sears K Klugar M Tufanaru C Leonardi-Bee J The revised JBI critical appraisal tool for the assessment of risk of bias for randomized controlled trials. JBI Evid Synth 2023;21(3):494-506. 36727247 10.11124/JBIES-22-00430

[32] Barker TH Habibi N Aromataris E Stone JC Leonardi-Bee J Sears K The revised JBI critical appraisal tool for the assessment of risk of bias quasi-experimental studies. JBI Evid Synth 2024;22(3):378-88. 38287725 10.11124/JBIES-23-00268

[33] Moola S Munn Z Tufanaru C Aromataris E Sears K Sfetcu R Chapter 7: Systematic reviews of etiology and risk. In: Aromataris E Lockwood C Porritt K Pilla B Jordan Z, editors. JBI Manual for Evidence Synthesis [internet]. JBI; 2024 [cited 2025 Jan 4]. Available from: https://synthesismanual.jbi.global.

